# Pyocyaneus Septicaemia

**Published:** 1913-09

**Authors:** 


Pyocyaneus Septicaemia. J. Michell (sic) Clarke, *Bristol Medico-Chir. Jour.*, March, 1913, reviews the literature which is quite extensive and includes both pure and mixed infections, and reports a pure case, in a laborer, aged 15, who died before autogenic vaccines could be used.

